# Comprehensive analysis of nucleocytoplasmic dynamics of mRNA in *Drosophila* cells

**DOI:** 10.1371/journal.pgen.1006929

**Published:** 2017-08-03

**Authors:** Tao Chen, Bas van Steensel

**Affiliations:** Division of Gene Regulation, Netherlands Cancer Institute, Amsterdam, The Netherlands; Technion, Israel Institute of Technology, ISRAEL

## Abstract

Eukaryotic mRNAs undergo a cycle of transcription, nuclear export, and degradation. A major challenge is to obtain a global, quantitative view of these processes. Here we measured the genome-wide nucleocytoplasmic dynamics of mRNA in *Drosophila* cells by metabolic labeling in combination with cellular fractionation. By mathematical modeling of these data we determined rates of transcription, export and cytoplasmic decay for 5420 genes. We characterized these kinetic rates and investigated links with mRNA features, RNA-binding proteins (RBPs) and chromatin states. We found prominent correlations between mRNA decay rate and transcript size, while nuclear export rates are linked to the size of the 3'UTR. Transcription, export and decay rates are each associated with distinct spectra of RBPs. Specific classes of genes, such as those encoding cytoplasmic ribosomal proteins, exhibit characteristic combinations of rate constants, suggesting modular control. Binding of splicing factors is associated with faster rates of export, and our data suggest coordinated regulation of nuclear export of specific functional classes of genes. Finally, correlations between rate constants suggest global coordination between the three processes. Our approach provides insights into the genome-wide nucleocytoplasmic kinetics of mRNA and should be generally applicable to other cell systems.

## Author summary

All mRNAs start from production in the nucleus, undergo exportation through nuclear pores and finally are degraded in the cytoplasm. A comprehensive characterization of the kinetic rates of all mRNAs is an important prerequisite for a global understanding of the regulation of the transcriptome and the cell. By conducting a time-series experiment and building a mathematical model, we trace the dynamics of mRNAs from the nucleus to the cytoplasm and determine the rates at each kinetic step at transcriptome-wide level. This information allows us to associate mRNA kinetic rates with a wealth of biological features and made some intriguing discoveries. We show mRNA decay is positively linked to transcript length while mRNA export is negatively linked to the length of the 3' UTR. We show binding of splicing factors is associated with faster rates of mRNA export. We provide evidence for global coordination between nuclear export an decay of mRNA. We show genes sharing specific functions tend to have similar nucleoplasmic kinetics, in which ribosomal proteins possessing special kinetic features exclusively stand out. Altogether, our integrated approach to quantitatively determine the rates of kinetic steps on a gene-by-gene basis provides a blueprint to obtain the global understanding of RNA regulation.

## Introduction

The production, nuclear export and degradation of mRNA are key steps in the control of cytoplasmic mRNA levels. Steady-state levels of transcripts in the cytoplasm are determined by the rates of these three processes. Hence, our understanding of gene regulatory systems requires quantitative knowledge of the relative contributions of each of these steps. Comparison of the kinetic rate constants for these steps across genes may provide insights into mechanisms of differential gene regulation.

Recent technological advances have enabled genome-wide measurements of mRNA dynamics [1, 2] and subcellular distribution [3]. In particular, the utilization of 4-thiouridine (4sU) as a reagent to metabolically label newly synthesized RNA has provided the means to monitor RNA dynamics with a minimal perturbation [1]. Using this approach, various fundamental kinetic rates of mRNA, such as synthesis, splicing and decay have been quantified at genome-wide level in a number of cell types from different species [4–9]. Global quantification of RNA kinetic rates has revealed at least four major biological insights: 1) different classes of genes utilize distinct kinetic strategies to sustain/alter their expression levels; 2) transcription is the primary determinant of the steady-state level of RNA/protein, with contributions much higher than degradation rates; 3) motif analyses and experimental approaches have identified a range of RNA binding proteins that regulate RNA stability [10, 11]; 4) the average rates of RNA decay differ dramatically between species.

These studies of mRNA kinetics have taken the total cellular mRNA as a single entity to calculate the overall turnover rate, overlooking nucleocytoplasmic transportation, which is thought to be a key aspect of mRNA dynamics and has been shown to be regulated by a variety of evolutionarily conserved mechanisms [12, 13]. Here we combined metabolic labeling of mRNA with cellular fractionation to systematically determine mRNA transcription, nuclear export and decay rates for thousands of genes. We developed a mathematical framework that infers nucleocytoplasmic kinetic rate constants from such labeling and fractionation time series data.

We chose *Drosophila* Kc167 cells as a representative model for metazoan cells, because of their ease to perform experiment and the availability of a wealth of genome-wide information. We report kinetic rate constants for 5420 genes and determine the relative contributions of each of transcription, nuclear export and decay to overall cytoplasmic abundance. Moreover, we uncover links between the three kinetic steps and transcript features, interactions of specific RNA-binding proteins and specific gene classes.

## Results

### Experimental design

To obtain genome-wide measurements of the nucleocytoplasmic dynamics of mRNA we followed a strategy as outlined in S1 Fig. Briefly, we performed a time series of metabolic labeling of RNA in *Drosophila* Kc167 cells using 4-thiouridine (4sU). We then isolated nuclear and cytoplasmic fractions from cells at each time point, and determined the relative abundance of “old” (unlabeled) mRNA for thousands of genes by high-throughput sequencing, as a function of time in both fractions. We then fed these measurements into a computational model that describes the process of sequential mRNA transcription, export and decay as a set of differential equations. Parameter fitting of the model to the measurements yielded kinetic rate constants for each of these three steps. Below we describe each step of the approach in more detail.

To label newly synthesized RNA we used 4sU, which is known to have no major effects on gene expression in *Drosophila* [14]. We further tested the impact of 4sU on gene expression of Kc167 cells by genome-wide comparison of mRNA expression levels between cells treated for 480 minutes with 4sU. The overall gene expression profile was not much affected by 4sU labeling (Spearman’s ρ = 0.98; P = 0) (S2A Fig). A small set of 59 genes that were influenced by 4sU labeling were excluded from subsequent analysis (S2A Fig). We then exposed cells to 4sU for 0, 30, 90, 180, 300, and 450 minutes and subsequently fractionated the cells into nuclear and cytoplasmic portions by hypotonic lysis and centrifugation. In each sample we spiked in a fixed amount of total RNA from the yeast *Saccharomyces cerevisiae* for normalization purposes, analogous to a previously reported approach [15]. The sum of nuclear and cytoplasmic portions showed very good genome-wide consistency with the unfractionated total transcriptome that was independently measured, indicating that the loss of RNA during fractionation was generally low (ρ = 0.89; P = 0; S2B Fig). We removed 107 genes for which this consistency did not hold up (S2B Fig).

We then purified pre-existing (i.e., unlabeled) RNA by removal of newly synthesized RNA through sulfhydryl conjugation and biotin-streptavidin pull-down [1]. Finally, we isolated poly-adenylated mRNA from the unlabeled fractions and subjected it to high-throughput sequencing. Because it is known that 4sU labeling shows a bias for long genes, we corrected for such bias as described previously [8]. From the changes in mRNA abundance in the two fractions over time we then inferred kinetic rate constants (see below).

We conducted these experiments as two biological replicates, and the reproducibility of the detected mRNA levels was high for all time points (S3A–S3D Fig). In bulk, the reads of both nuclear mRNA and cytoplasmic mRNA showed a continuous decrease over time relative to the yeast spike-in, reflecting the expected replacement of unlabeled mRNA by labeled mRNA (S3E Fig). The amount of unlabeled mRNA eventually asymptotes to a plateau of 7.3±1.2% (S3F Fig; Methods), which may reflect a pool of highly stable transcripts or incomplete removal of labeled mRNA. In order to check the purity of the nuclear and cytoplasmic fractions we monitored intron:exon ratios for a number of transcripts by quantitative reverse transcription polymerase chain reaction (qRT-PCR). This revealed predominant presence of introns in the nuclear fraction, as expected (S3G and S3H Fig). Furthermore, analysis of the high-throughput sequencing reads indicated a substantial enrichment (9.3±2.0 fold) of rRNA in the cytoplasmic fraction (S3I Fig). These results indicate that our measurements of pre-existing mRNA abundance over time in both the nuclear and the cytoplasmic compartments were generally robust.

### Quantitative modeling

**Fig 1:**
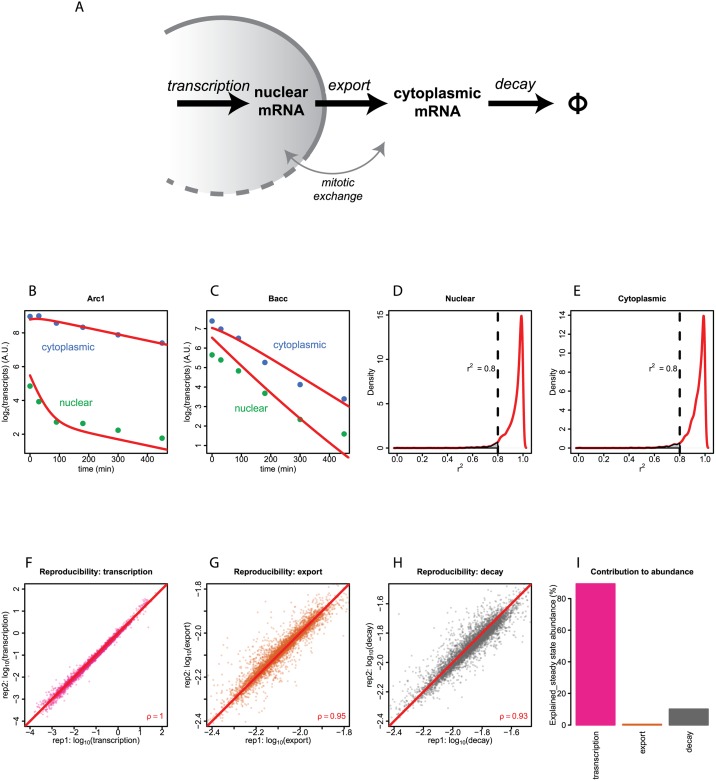
Mathematical modeling of the nucleocytoplasmic dynamics of mRNA. **(A)** Schematic illustration of the kinetic steps in the model. **(B, C)** Fitting of the model to experimental data for two example genes (*Arc1* and *Bacc*). Green dots and blue dots represent nuclear and cytoplasmic transcript abundance, respectively, normalized to yeast spike-in. Red curves depict the fitted kinetic model. **(D, E)** Global distribution of the goodness of fit scores for nuclear and cytoplasmic fractions, respectively, assessed by coefficient of determination (r^2^). Only genes with r^2^ > 0.8 are used for downstream analyses. **(F, G, H)** Scatter plots showing the reproducibility of modeled rates of transcription, export and cytoplasmic decay of two biological replicates. Each dot represents one gene. Red lines indicate the perfect diagonals. **(I)** Contributions of the variance of the rates of transcription, export and cytoplasmic decay to the variance of steady state transcript abundance.

Subsequently, we applied mathematical modeling to the time series measurements to estimate rates of transcription, nuclear export and cytoplasmic decay for each transcript. We designed a set of first-order ordinary differential equations to describe the nucleocytoplasmic dynamics (Fig 1A; see Methods). We assumed a steady-state model of a pool of non-synchronously dividing cells in which mature transcripts are produced in the nucleus, transported to the cytoplasm, and degraded in the cytoplasm, with each step described by a first-order reaction rate constant. In this model we assumed that transport of mRNA across the nuclear pore complex is unidirectional, which is generally supported by previous studies [12, 13, 16–19]. However, because transcripts redistribute between the nuclear and cytoplasmic compartments in the period between nuclear envelope breakdown and reformation during mitosis, we included kinetic terms to model this process (Fig 1A). Furthermore, we followed the prevailing model that degradation of polyadenylated mRNA occurs predominantly in the cytoplasm [20, 21]. Lastly, because it is not feasible to accurately quantify the abundance of every alternative transcript, we combined sequence reads from alternative transcripts, yielding a single kinetic model for each gene. An analogous mathematical framework based on similar assumptions was reported recently [22].

For each gene, we fitted this model to the experimental data from each of the two biological replicates (S1 Table). As examples, we show the fitting results of the genes *Arc1* and *Bacc* (Fig 1B and 1C). Genome-wide, the goodness of fit was high, as indicated by the coefficients of determination (*r*^2^) globally being close to 1 for both nuclear and cytoplasmic compartments (Fig 1D and 1E). For subsequent analyses, out of 5,730 genes that were detected in all the time points of measurements after correction for 4sU labelling bias and exclusion for 4sU labeling and fractionation influence, we retained 5420 genes that have *r*^2^ > 0.8. To test whether the data are compromised by undersampling, we down-sampled the sequencing reads to only 25 percent and repeated the modeling. The resulting estimated kinetic rates are generally consistent with the results based on the full dataset, demonstrating the robustness of the modeling (S4A–S4C Fig).

Analysis of the genes that did not fit the first order kinetic model well (*r*^2^ < 0.8) indicated that they have substantially lower transcription rates (S5A Fig). This could mean that the modeling is less accurate at low expression levels. However, genes with relatively poor fits also tend to have long introns (S5B Fig); we speculate that processing of these mRNAs is more complex and cannot be captured accurately by our computational model. Importantly, for the set of 5,420 genes that have *r*^2^ > 0.8, all three modeled parameters have very good reproducibility between the two replicates (Fig 1F–1H), ruling out overfitting as the cause for the good agreement between the modeled values and experimental data. We therefore used these 5,420 genes for subsequent biological analysis.

Due to normalization to the spiked-in yeast mRNA, transcription is expressed in arbitrary units per minute. We emphasize that these transcription rates refer to the speed of production of polyadenylated mature mRNA, not the distance travelled by RNA polymerase as a function of time. The rates of export and cytoplasmic decay are expressed as fraction per minute, with a median value of 0.83% per minute (corresponding to a half-life of 1.4 hours), and 1.40% per minute (a half-life of 0.8 hours), respectively.

### Contributions to steady-state mRNA abundance

For the majority of genes, the rate constants of transcription, export and cytoplasmic decay span about 5, 0.5 and 0.9 orders of magnitude, respectively (Fig 1F–1H). This prompted us to calculate the relative contributions of the three processes to the genome-wide variance in steady-state mRNA abundance. The results (Fig 1I) indicate that transcription explains most of the variance (89.4%), while the contribution of cytoplasmic decay is lower but still substantial (10.1%). In contrast, the contribution of nuclear export to the genome-wide variance is negligible (0.5%). Our estimation of the relative contribution of mRNA decay to the steady state mRNA abundance is lower than previously estimated for yeast (~30%) [8], but higher than estimated for mouse embryonic stem cells (~1.4%) [23].

### Kinetic rates are related to transcript length

**Fig 2:**
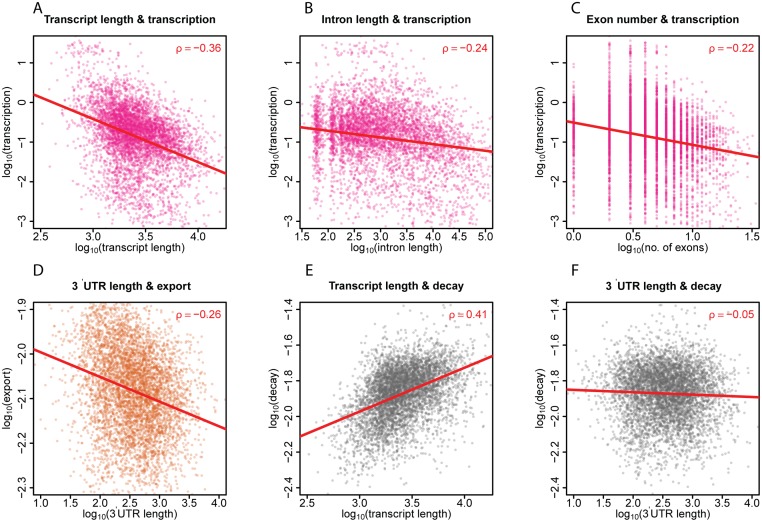
The relationships between transcript length and kinetic rates of mRNA. **(A, B, C)** Scatterplots showing the relationships between transcript length, intron length, number of exons and transcription rates on logarithmic scales. Red lines indicate fitting by linear regression. Spearman (ρ) correlations are indicated. **(D)** Relationship of 3’UTR length and export rates. **(E, F)** Correlations between transcript and 3’UTR length, respectively, and decay rates.

Next, we sought to identify potential determinants of individual kinetic steps. First, we investigated a possible relationship between transcript length and kinetic rates. This revealed that transcription rate has a considerable negative correlation with mRNA length (ρ = -0.36; P = 1.1E-164; Fig 2A), suggesting that mature transcripts are generally less efficiently produced from long genes than from short genes. In part, this may be explained by a more extensive (co-transcriptional) splicing of long transcripts. Indeed, we find that transcription rate is negatively correlated with intron content (ρ = -0.24; P = 1.1E-69; Fig 2B) and with the number of exons (ρ = -0.22; P = 1.8E-60; Fig 2C). This is in agreement with observations that elongation tends to slow down at exons [24] and that transcribed length has negative relationship with the rapidity of RNA polymerase II (Pol II) recruitment [25].

Export rates show no correlation with total mRNA length (ρ = 0.01; P = 0.6), suggesting that mRNA length is not a major limiting factor for transport through the NPC. However, export rate does show a notable negative correlation with the length of the 3’ UTR (ρ = -0.26; P = 4.6E-85; Fig 2D) and to a much lesser extent with the length of the coding region (ρ = 0.13; P = 3.2E-22) or 5' UTR (ρ = -0.12; P = 4.3E-18). We speculate that the binding of regulatory proteins to the 3’ UTR may slow down export or actively retain transcripts in the nucleus.

Interestingly, cytoplasmic decay rate shows a considerable positive correlation with total mRNA length (ρ = 0.41; P = 3.8E-215; Fig 2E). We propose two possible explanations for this surprising link. First, cytoplasmic mRNA degradation may be initiated by stochastic attack by an endonuclease. In this model, long mRNAs simply have a higher probability to be cleaved than short mRNAs. The decay rate generally scales sub-linearly with mRNA length, as indicated by a linear regression slope of 0.25 in log-log space (Fig 2E). This may reflect that mRNA is generally folded in a three-dimensional ribonucleoprotein particle, and the proportion of the mRNA that is buried inside this particle may increase with the linear length. A second, not mutually exclusive explanation may be that long mRNA molecules are more likely to contain motifs that have affinity to proteins that target the mRNA to the cytoplasmic decay machinery. Interestingly, decay rate has virtually no correlation with the length of the 3’UTR (ρ = -0.05; P = 8.2 E-4; Fig 2F) which is the primary site where miRNAs act [26, 27]. miRNA-directed degradation may therefore not be the chief mechanism for cytoplasmic decay in *Drosophila* Kc167 cells.

### Connections between RNA-binding proteins and nucleocytoplasmic mRNA kinetics

**Fig 3:**
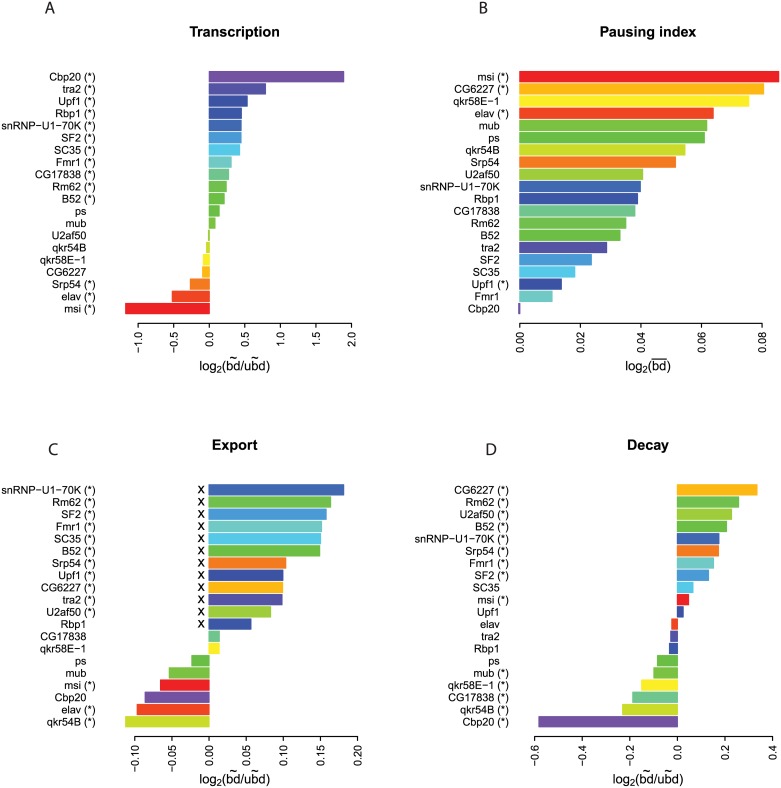
Links between binding of RBPs and kinetic rates of mRNA. **(A)** Log_2_-transformed ratios of median transcription rates of genes bound ($\tilde{bd}$) and unbound ($\tilde{ubd}$) by RBPs as indicated. **(B)** Log_2_-transformed mean Pol II pausing indices of transcripts bound by each RBP ($\bar{bd}$). **(C)** Same as (A), but for export rates. RBPs with roles in splicing are marked by "x". **(D)** Same as (A), but for decay rates. RBPs in all the panels are ranked according to their associated values, but colors are same as in **(A)**. RBP binding data was taken from [31]. RBPs of which binding is significantly associated with rates of transcription **(A)**, pausing index (**B**), export **(C)** and cytoplasmic decay **(D)** are marked by (*) (P < 0.01, two-sided Wilcoxon test, adjusted by the Holm—Bonferroni method).

RNA-binding proteins (RBPs) are well known for their regulatory roles in specific steps of RNA metabolism [28–30], but genome-wide assessment has not been yet carried out. We took advantage of recently published transcriptome-wide RNA interaction profiles of 20 RBPs [31] to uncover putative links with kinetic properties of mRNA. The interaction profiles were generated from *Drosophila* S2 cells, which are similar to the Kc167 cells that we used in our study. We compared the median kinetic rates of mRNAs that are bound and not bound by each RBP (Fig 3A, 3C and 3D). This revealed that about two-thirds of the RBPs are significantly correlated with each step of kinetic regulation. The differences range from ~8.4 fold for transcription, to ~1.2 fold for export and ~1.9 fold for cytoplasmic decay. Overall, the correlations between RBP binding and the rate constants were similar for long and short transcripts (S6A–S6F Fig), indicating that transcript length is not a substantial confounding factor in this RBP analysis.

The RBP that is associated with the highest transcription rate is Cbp20, a key component of the nuclear cap-binding complex (Fig 3A). Its human homolog has previously been reported to facilitate Pol II release from promoters through interaction with transcription elongation through P-TEFb [32], which should lead to reduction of the fraction of paused Pol II. We investigated this by computing the ‘pausing index’ of Pol II for each gene [33]. This pausing index is generally inversely correlated with transcription rates, and we found Cbp20-bound transcripts to have the lowest pausing index (Fig 3B). It thus is likely that Cbp20 also has a role in releasing paused Pol II in Kc167 cells. The Exon Junction Complex protein Upf1 that links to nonsense-mediated decay also correlates with high transcription (Fig 3A) and a relatively low pausing index (Fig 3B). A previous study in *S*. *cerevisiae* indicated that RNA decay factors can boost transcription independent of their function in RNA degradation [34].

The RBPs that are associated with the slowest transcription rate are msi and elav, which have little overlap in their mRNA binding specificities (S6J Fig). However, these two proteins are expressed at extremely low levels in Kc167 cells [35]. It is therefore unlikely that they act as repressors of transcription. We speculate that their target mRNAs may in fact require binding of elav/msi for efficient transcription, and thus may be transcribed at higher levels in the cell types where these proteins are present (e.g., nervous system cells). We tested this hypothesis by comparing the expression level of transcripts in Kc167 that were shown to be bound by the ectopically expressed msi and elav [36], to that of ML-DmBG2-c2, a cell line representing cells of the central nervous system where these two proteins are expressed. Indeed, those genes that can be bound by msi and elav show statistically higher expression in ML-DmBG2-c2 cells (msi: P = 1.9e-7; elav: P = 2.2e-4; two-sided Wilcoxon test). This suggest that msi and elav can promote transcription in the corresponding tissues, but further experimental evidence is needed.

Remarkably, we found that all the 12 RBPs known to be involved in splicing [31] are associated with somewhat higher export rates. These RBPs, which tend to have overlapping mRNA binding specificities (S6J Fig) occupy the top 12 positions when ranked by mean export rate (Fig 3C). This is in agreement with previous reports that have linked splicing factors to nuclear export [37, 38]. In particular, among the splicing factors, SF2 has been shown as the adapter protein for TAP-dependent mRNA export [39]. Paradoxically, we find that mRNAs from intron-containing genes are generally not more rapidly exported than mRNAs from intron-less genes; there is even a slight opposite trend (S6G Fig), which may be due to the fact that intronless genes generally have shorter 3’UTR (S6H Fig), which in turn is associated with higher export rate (Fig 2D). Analysis of the published RBP binding data [31] indicates that transcripts from genes with introns are not enriched for the binding of splicing-related RBPs, compared to transcripts from genes lacking introns (S6I Fig). Together, these data suggest that splicing factors may promote export of mRNA at least in part independently of their role in splicing.

The RBP that is associated with the highest cytoplasmic decay rate is CG6227, a putative DEAD-box containing RNA helicase with so far unknown function (Fig 3D). Lastly, the factor that is associated with the slowest cytoplasmic decay rate is Cbp20. Transcripts with a 7-methylguanosine cap are thought to be bound by Cbp20. This protein is generally restricted to the nucleus, while other proteins take over the cap-binding function in the cytoplasm [40]. Although it is not confirmed that this is also the case in *Drosophila*, it is therefore unlikely that Cbp20 directly affects cytoplasmic decay. Rather, Cbp20 binding to mRNA as detected in a total cell lysate [31] probably reflects the presence of 7-methylguanosine on the transcripts, and this capping is regulated in Drosophila cells and known to inhibit cytoplasmic decay [41] [42]. In conclusion, this analysis identifies candidate proteins that may control specific steps of nucleocytoplasmic mRNA kinetics.

### Chromatin links to kinetic regulation

Chromatin is well-known for its role in regulating transcription, but there is also evidence that it may control the downstream fate of RNA. For example, specific histone modifications can influence alternative splicing [43, 44] and promoter-bound proteins can direct cytoplasmic mRNA stability [45] [46]. We therefore asked whether the kinetic parameters derived from our measurements are correlated with the chromatin environments of the genes. To this end we stratified these parameters by the previously characterized five principal chromatin states [47] at both the transcription start sites (TSSs) and transcription termination sites (TTSs).

**Fig 4:**
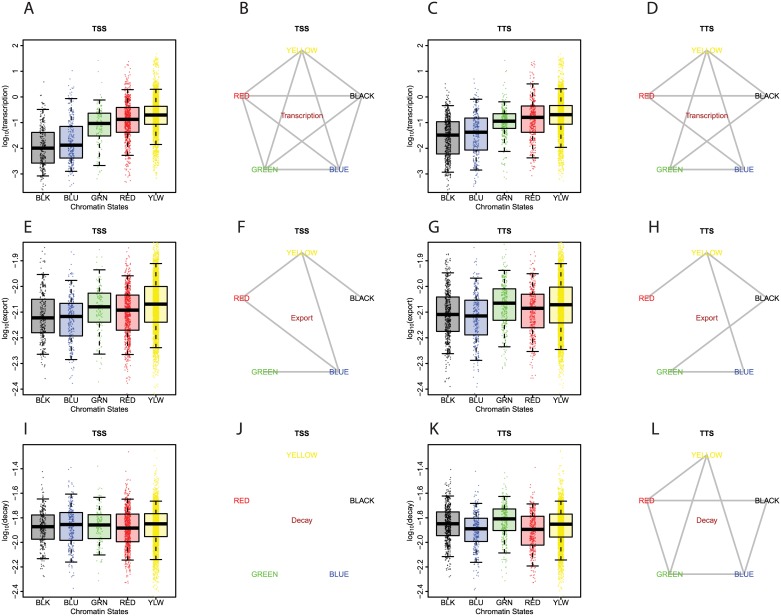
Associations of chromatin states and kinetic rates of mRNA. Links between chromatin states and transcription rates: **(A)** transcription rates of genes divided by their chromatin states at their transcription start sites (TSS). Whiskers represent the 5th percentile and the 95th percentile; **(B)** Between each pair of chromatin states at TSS, a line is drawn if there is statistical difference (p <0.01, Tukey’s range test, ANOVA); **(C, D)** same as **(A, B)**, but for chromatin states at transcription termination sites (TTS). Similar analyses are displayed for nuclear export **(E-H)** and cytoplasmic decay **(I-L)**.

As expected, the modeled transcription rates differ widely among chromatin states, both at TSSs and TTSs (Fig 4A–4D). BLUE chromatin, which is marked by H3K27me3 and Polycomb proteins, is associated with very low transcription rates. This is consistent with a wide body of literature indicating that Polycomb complexes directly repress transcription [48]. BLACK chromatin, a hitherto poorly characterized repressive chromatin type that carries H3K27me2 but not Polycomb proteins [14, 47, 49], shows similarly low transcription rates, suggesting that BLACK chromatin also acts at the level of transcription. GREEN chromatin, marked by H3K9me2 and HP1 shows intermediate transcription rates, while the euchromatic YELLOW and RED states show high transcription activity, as expected.

We also observed modest correlations between chromatin states and post-transcriptional kinetic parameters. For both TSS and TTS, transcripts arising from BLACK and BLUE chromatin showed lower export rates than those from YELLOW and GREEN chromatin (Fig 4E–4H), but the difference is only ~1.1-fold. For cytoplasmic decay, we also observed significant chromatin-state-associated differences (Fig 4I–4L), but only at TTSs and of minor magnitude (up to ~1.2 fold). Notably, HP1-containing GREEN chromatin is associated with highest decay rates, which is consistent with a previous study that demonstrated that HP1 mediates heterochromatic transcript decay in *S*. *pombe* [50].

We obtained similar results with a 9-state chromatin state model, which is mostly based on histone modification maps [51] (S7 Fig). In particular state 6, roughly equivalent to BLUE chromatin, shows associations with low transcription and low export, especially when present at TSSs. Other states correlate with transcription levels but show only minor differences in export and decay rates. Together, these results indicate that chromatin states primarily affect transcription, and may have intriguing but subtle links to mRNA export and decay.

### Relationships between the kinetic rates of mRNA

**Fig 5:**
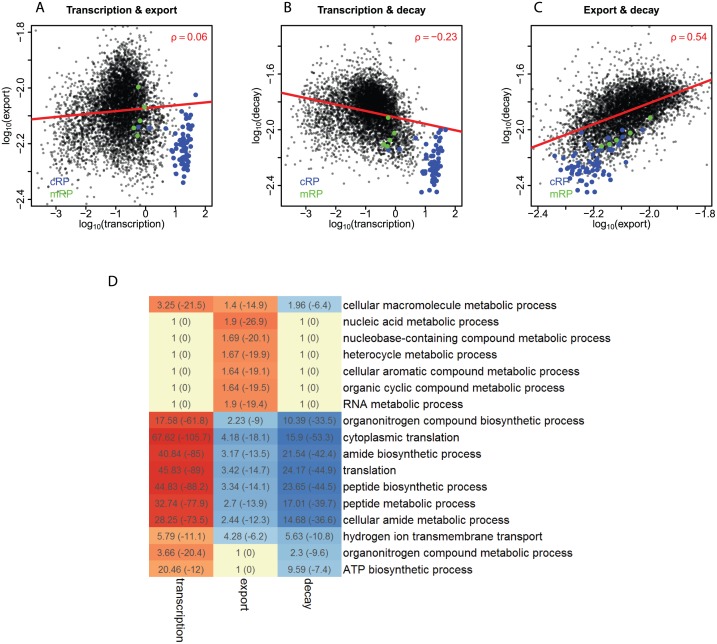
Links between kinetic rates; and GO analysis. **(A, B, C)** Scatterplots showing pairwise relationships between the rates of transcription, export and cytoplasmic decay on logarithmic scales. Spearman (ρ) coefficients are indicated. Red lines indicate fitting by linear regression. Genes encoding cytoplasmic ribosomal proteins (cRP) are marked in blue and genes encoding mitochondrial ribosomal proteins (mRP) are marked in green. **(D)** Gene ontology analysis. Heatmap showing associations between kinetic rates and "biological process" GO categories. Processes are shown if they are enriched for at least one kinetic rate with p < 10^−11^. The enrichment scores of genes with the respective GO terms are indicated, with log_10_-transformed p-values in parentheses, according to GOrilla [82]. Red color marks enrichment for high kinetic rates while blue color marks enrichment for low kinetic rates. See also S2 Table.

Cross-talk has previously been observed between mRNA transcription and decay in yeast [3, 9, 34, 52]. This prompted us to analyze possible relationships between the kinetic rate parameters in our data. Pairwise scatterplots revealed a number of interesting patterns. First, there is virtually no correlation between the rates of transcription and export (ρ = 0.06, P = 3.6E-05, Fig 5A). Second, a moderate negative relationship exists between the rates of transcription and cytoplasmic decay (ρ = -0.23, P = 5.3E-66, Fig 5B), which is in part due to a separate group of genes that we will discuss below. Third, we observe a positive relationship between rates of export and cytoplasmic decay (ρ = 0.54, P ≈ 0, Fig 5C). These data suggest global coordination between mRNA decay and both transcription and nuclear export. The underlying mechanism is unclear; in yeast two subunits of RNA polymerase II have been implicated in such coordination [53–55].

### Classes of genes with distinct mRNA kinetics

One group of genes stands out in the scatterplots (blue dots, Fig 5A–5C). Virtually all genes in this group encode for cytoplasmic ribosomal proteins (cRPs). These genes are characterized by very high transcription, fairly low export, and very low cytoplasmic decay (Fig 5A–5C). It is noteworthy that the cRP genes are completely separated from nuclear genes encoding mitochondrial ribosomal proteins (mRPs), which also cluster in the scatterplots but show less extreme values (green dots in Fig 5A–5C). The basis for this unique regulation is unclear, but we speculate that the 5'-terminal oligopyrimidine tract (TOP) motif, which is found in most cRP mRNAs [56, 57], plays a role in this.

We searched for other functional categories of genes with distinct kinetic parameters by gene ontology (GO) analysis (Fig 5D, S2 Table). Translational machinery genes, primarily comprising of cRP genes, exhibit relatively high transcription, low export and low cytoplasmic decay, as expected. Genes that are responsible for primary metabolic processes are highly transcribed, highly exported and slowly degraded, representing prominent efficiency of expression. The other house-keeping genes, including various synthetic and transport activities of macromolecules, are highly transcribed and lowly decayed but do not possess characteristic export rates. Lastly, it is intriguing that genes linked to neural differentiation and cellular response to stress are enriched for high export, and the latter process is also enriched for high decay. Presumably this provides routes to activate or inactivate gene regulatory cascades in a rapid and flexible manner when extrinsic stimuli are received in developmental processes or stress response. We note that genes with low transcription or high decay may be under-represented in this GO analysis, because they are less likely to have passed our stringent filters for model fitting. Overall, these results reveal curious links between specific functional gene modules and kinetic properties of their transcripts.

## Discussion

By combination of metabolic labeling, cell fractionation and mathematical modeling we determined key parameters of the nucleocytoplasmic dynamics of mRNA for 5,420 genes in *Drosophila* cells. Our subsequent analyses revealed that these kinetic rates are linked to various molecular components, have relationships with each other and are linked to specific biological processes.

The export and cytoplasmic decay rates deduced from from our measurements and modeling are both on average in the range of ~1% per minute. These values are generally similar to rates estimated by previous studies. Genome-wide studies have determined median mRNA decay rates to range from 1.4% (*H*. *sapiens*, [58]), 0.8% (*M*.*musculus*, [6]), to 6.3% (*S*. *cerevesiae*, [8]), while focused analyses of individual transcripts have yielded values ranging from ~1% per minute in zebrafish [59] to 1.67% per minute in mouse tissues [22], which is very similar to our estimates. For nuclear export, recent microscopy studies estimated the retention time of mRNAs in human cells to be about 40–60 minutes, which corresponds to a rate of 1.2%-1.7% per minute [60], and 8.6 minutes (~8% per minute) in mouse tissue [22]. The latter export rate is somewhat higher than we typically observed, which may be explained by differences in cell type, species, or techniques used. Our estimates of transcription rates are in arbitrary units and can therefore not be compared to other studies.

Our results indicate that nuclear export generally has a relatively minor impact on steady-state mRNA levels. Nevertheless, links with 3'UTR length and the binding patterns of RBPs point to mechanisms that regulate mRNA export. This is in line with previous gene-specific studies indicating that sequences in the 3'UTR of mRNA can affect nuclear export [61, 62]. Our observation that several functional classes of genes show higher or lower export rates points to a certain degree of coordination of the export of mRNAs belonging to the same pathway. Some of the processes that we identified involve responses to DNA damage, stress and nutrients, as well as differentiation. This extends previous observations that the export of individual transcripts can be under control of such signaling events [62–67]. It will be interesting to study the changes in global nucleoplasmic kinetics of mRNAs when these pathways are activated by the appropriate stimuli.

It is also noteworthy that transcripts derived from genes bound by Polycomb complexes (BLUE chromatin) show slightly slower export rates. We speculate that this may be caused by the broad affinity of Polycomb complexes for RNA [68, 69], which may lead to some sequestration of transcripts in the nucleus. This may reduce the availability of the transcripts in the cytoplasm to some degree. Another interesting possibility is that temporary nuclear retention of transcripts may buffer bursts of transcription [22].

We observed that the rate of cytoplasmic decay is positively correlated with transcript length. This is somewhat surprising, because it is generally thought that degradation of mRNA is primarily mediated by the 3' exonuclease activity of the exosome [8, 70–72], which is unlikely to lead to a faster decay for a longer RNA. Rather, the positive correlation with transcript length points to decay mediated by stochastic activity of an endonuclease [73]. A candidate for such endonuclease activity is the *Drosophila* exosome subunit Dis3, which harbors ribo-endonuclease activity and is expressed in Kc167 cells [74].

In summary, we outlined a generally applicable experimental strategy and a mathematical framework to determine important parameters of nucleoplasmic dynamics for thousands of genes. One possible extension of our strategy is to quantify both the unlabeled and the 4sU-labeled mRNA fractions over time, rather than the unlabeled fractions alone. This may provide an even more precise view of the nucleoplasmic kinetics, particularly of transcripts with high transcription and export rates. The dataset reported here–as well as the uncovered links with mRNA characteristics, RBPs, and chromatin states–provides a foundation to begin to untangle the underlying mechanisms. Application of this approach to other cell types and species will help to understand the global principles of mRNA regulation in the context of differentiation and evolution.

## Materials and methods

### Cell culture

*Drosophila* Kc 167 were cultured as previously described [75].

### Metabolic labeling and hypotonic fractionation

Around 1 million *Drosophila* kc167 cells were separately labelled in 5 ml medium in 10 cm culture dishes with 300 μM 4-thiouridine (Sigma-Aldrich, Cat No. T4509) for 0, 30, 90, 180, 300, 450 minutes. Cells were spun down, washed with serum-free medium and suspended with 120 μl hypotonic buffer consisting of 10mM NaCl, 2mM MgCl, 10mM Tris-HCL (pH = 7.8), 5mM dithiothreitol (DTT), 0.5% nonylphenoxypolyethoxylethanol (NP-40). Suspensions were put on ice for 5 min and spun down at 2000 g at 4 degree for 5 minutes. Supernatants were taken out as cytoplasmic fraction and the pellets were suspended in 120μl hypotonic buffer as nuclear fraction. 700 μl TRIsure (BIOLINE, Cat No. BIO-38032) containing 1 ng/μl total RNA from *S*. *cerevisiae* as spike-in was added to both fractions and RNA extractions were performed following the protocol of a published study [1].

### Examination of the purity of hypotonic fractionation

1 μg of nuclear and cytoplasmic RNA samples were reverse-transcribed (BIOLINE, Tetro reverse transcriptase, Cat No. BIO-65050) with random-hexamers (BIOLINE, Cat No. BIO-38028). The reaction was subsequently diluted 20 times with water, 4 μl of which was used for qPCR. The primers used are:

Lam exon forward, GAAGACCTGAATGAGGCGCT; Lam exon reverse, TGGTGTTCTCCAGGTCAACG; Lam intron forward, AAGTGCGTGGAAACTGAATCG; Lam intron reverse, CTTGCTTGAAACCACGCCTT; Fmo-2 exon forward, TGATGCAGTGCTTCCACAGT; Fmo-2 exon reverse, ATGTTCTGCACCGGCTACAA; Fmo-2 intron forward, GGCCCCGTGAGATCGATTAG; Fmo-2 intron reverse, TGGTAGCGACGTCACGTATT.

### Isolation of pre-existing RNA and total RNA

Nuclear and cytoplasmic RNA were labeled with EZ-Link^™^ HPDP-Biotin (ThermoFisher Cat No. 21341) and pre-existing RNA were purified by removal of biotinylated newly-synthesized RNA as previously described [1]. Total RNA was directly extracted from unfractionated cells following the protocol of a published study [1].

### RNA sequencing

Polyadenylated RNA was purified by oligo-dT beads for both nuclear and cytoplasmic fractions, reverse transcribed (SuperScript II Reverse Transcriptase, Invitrogen, # 18064–014) and constructed into strand-specific libraries using the TruSeq Stranded mRNA sample preparation kit (Illumina Inc., San Diego, RS-122-2101/2) according to the manufacturer's instructions (Illumina, # 15031047 Rev. E). The generated cDNA fragments were 3' end adenylated and ligated to Illumina Paired-end sequencing adapters and subsequently amplified by 12 cycles of PCR. The libraries were analyzed on a 2100 Bioanalyzer using a 7500 chip (Agilent, Santa Clara, CA), diluted and pooled equimolar into a 12-plex for each replicate and subjected to sequencing with 50 base single reads on a HiSeq2500 using V4 chemistry (Illumina Inc., San Diego). The two replicates were sequenced in two separate lanes. The total reads for the two replicates are 182,747,266 and 177,771,796, with even reads distribution for each time point.

Reads were mapped first to the transcriptome of *S*. *cerevisiae* (Saccharomyces_cerevisiae.R64-1-1.78) and then to the transcriptome of *D*. *melanogaster* (Drosophila_melanogaster.BDGP5.77) by Tophat [76].

### Calculation of relative abundance of transcripts

For each time point, the number of reads that were mapped to *Drosophila* transcriptome was divided to the number of reads that were mapped to Saccharomyces transcriptome to obtain the factor for normalization. The number of Fragments Per Kilobase of transcript per Million mapped reads (FPKM) for each gene was calculated using the default setting of Cufflinks [77] and multiplied by the factor for normalization to obtain the relative abundance of transcripts.

### The cutoff function to remove outliers

We intended to exclude genes that show strong anomalous behaviors which may be due to 4sU labeling or the fractionation procedure. Instead of linear exclusion, we also took the non-uniform distribution of dispersion into account. Considering two transcriptomes x and y of comparison, we used a simple non-linear function to depict the dispersion:

$$\text{f}\left(\text{x}\right)=\text{sd}\left(\text{y}\right)=\text{m}+\text{n}e^{-px}$$

where x is the RNA abundance and f(x) is deviation from the perfect diagonal which corresponds to the standard deviation of y. The abundance of transcripts in the data were divided into 1000 intervals and the corresponding values were calculated. The non-negative parameters m,n,p were then fitted by the Levenberg—Marquardt algorithm using the ModFit function in the package of FME in R. We defined outliers as genes that exceed twice the amount of technical dispersion:

outliers∣{y>x+2f(x) or x>y+2f(y)}

### Correction for 4sU labelling bias

The labelling bias of 4sU as function of the length of genes was corrected previously described [8]. The correction factor is calculated as

$$\text{F}=1-{\left(1-\text{pr}\right)}^{\text{Nu}}$$

where pr is the labeling probability that is determined to be around 0.01 [8] and Nu is the number of uridine in the transcripts of individual genes.

### Modeling remaining 4sU labeled transcripts after biotin-streptavidin removal

To calculate the magnitude of remaining newly-synthesized RNA that contains 4sU after streptavidin removal, we assume first order turnover of total transcripts (detailed in the next section of Quantitative modeling), and add a term for the remaining fraction of 4sU (C) that has not been removed by streptavidin pulldown, similar to a previous approach [6].

Using the notations from the next section of Quantitative modeling, the abundance of newly synthesized RNA over time is

$$W_{4sU}\left(\text{t}\right)={\text{W}}_{0}({\text{e}}^{\text{gt}}-{\text{e}}^{-{\text{ k}}_{\text{T}}\text{t}})$$

Considering potential contamination factor (U) of *W*_*4sU*_ into the unlabeled fraction, and assuming the pre-exsiting RNA follows first order turnover, the pre-existing fraction P is

$$P\left(t\right)=\;\left({\text{W}}_{0}-U\cdot W_{4sU}\left(t\right)\right){\text{e}}^{-{\text{ k}}_{\text{T}}\text{t}}=W_{4sU}\left(\text{t}\right)={\text{W}}_{0}(1-U{\text{e}}^{\text{gt}}+U{\text{e}}^{-{\text{ k}}_{\text{T}}\text{t}}){\text{e}}^{-{\text{ k}}_{\text{T}}\text{t}}$$

And the contamination fraction R(t) is

$$R\left(t\right)=\;U\cdot W_{4sU}\left(t\right)=U{\text{W}}_{0}({\text{e}}^{\text{gt}}-{\text{e}}^{-{\text{ k}}_{\text{T}}\text{t}})$$

Therefore, the expected unlabeled fraction with contamination is

W(t)=P(t)+R(t)

The contamination factor U is estimated by minimizing

$$d=\frac{1}{\text{n}}\sum_{i=1}^{n}{\left(L(W_{m}\left(t_{i}\right)log\frac{W_{m}\left(t_{i}\right)}{W\left(t_{i}\right)}\right)}^{2})$$

Where L is the loess function described in the Eq (18) of the next section and *W*_*m*_(*t*_*i*_) is the measured abundance at a time point i. We used the Levenberg—Marquardt algorithm to fit experimental data using the ModFit function in the package of FME in R. U is estimated to be (7.3 ±1.2) %.

### Quantitative modeling of kinetic rate constants

The non-compartmentalized overall mRNA dynamics of non-synchronized Kc167 cells can be described by a simple ordinary differential equation

$$\frac{dW\left(t\right)}{dt}=k_{S}-k_{T}W\left(t\right),$$ (1)

whereby for a given gene, W stands for the total amount of bulk mRNA with the unit of Fragments Per Kilobase Of Exon Per Million Fragments Mapped (FPKM), t for time in minutes (min), *k*_*s*_ for transcription rate in FPKM·min^-1,^ *k*_*T*_ for overall turnover rate in min^-1^. The equation satisfies the quasi-steady-state assumption, i.e.,

$$\frac{dW\left(t\right)}{dt}=gW\left(t\right),$$ (2)

since in standard culture medium, cells in the dish do nothing but merely doubling. Let g be the proliferation rate, which can be calculated from the measured doubling time of 24 hours of kc167 cells,

$$g=\frac{100\;ln2}{24*60}\%=0.048\%{\text{min}}^{-1},$$ (3)

For pre-existing RNA, total amount *W*_*p*_ satisfies,

$$\frac{dW_{p}\left(t\right)}{dt}=-\;k_{T}W_{p}\left(t\right),$$ (4)

Denoting *W*_*p*_(0) by *W*_0_,

$$W_{p}\left(t\right)=W_{0}e^{-\;k_{T}t}.$$ (5)

Similarly, the simplest model for the nucleocytoplasmic dynamics of mRNA can be written as

$$\left(\begin{array}{c} \frac{dN\left(t\right)}{dt}\\ \frac{dC\left(t\right)}{dt} \end{array}\right)=\left[\begin{array}{c} k_{S}-\;(k_{E}+k\prime_{f})\\ \;(k_{E}+k\prime_{f}) \end{array}\begin{array}{c} k_{f}\\ -\left(k_{D}+k_{f}\right) \end{array}\right]\left(\begin{array}{c} N\left(t\right)\\ C\left(t\right) \end{array}\right)=g\;\left(\begin{array}{c} N\left(t\right)\\ C\left(t\right) \end{array}\right),$$ (6)

whereby for a given gene, *N and C* stand for the total amount of mRNA in the, correspondingly, nuclear and cytoplasmic compartments in FPKM. For post-transcriptional kinetic rates, *k*_*E*_ stands for exportation rate from the nucleus to the cytoplasm, *k*_*D*_ for cytoplasmic decay rate, and *k*_*f*_ stands for cytoplasm-to-nucleus inward transfer rate while $k_{f}^{\prime }$ for nucleus-to-cytoplasm outward transfer rate during mitosis, which will be discussed later. All post-transcriptional kinetic rates are in the unit of min^-1^. The equation also satisfies the quasi-steady-state assumption, at t = 0, denoting *N*(*t*) by *N*_0_ and *C*(*t*) by *C*_0_,

$$\left[\begin{array}{c} k_{S}-\;(k_{E}+k\prime_{f})\\ \;(k_{E}+k\prime_{f}) \end{array}\begin{array}{c} k_{f}\\ -\left(k_{D}+k_{f}\right) \end{array}\right]\left(\begin{array}{c} N_{0}\\ C_{0} \end{array}\right)=g\;\left(\begin{array}{c} N_{0}\\ C_{0} \end{array}\right).$$ (7)

For pre-existing *N*_*p*_ *and C*_*p*_,

$$\left(\begin{array}{c} \frac{dN_{p}\left(t\right)}{dt}\\ \frac{dC_{p}\left(t\right)}{dt} \end{array}\right)=\left[\begin{array}{c} -\;(k_{E}+k\prime_{f})\\ \;(k_{E}+k\prime_{f}) \end{array}\begin{array}{c} k_{f}\\ -\left(k_{D}+k_{f}\right) \end{array}\right]\left(\begin{array}{c} N_{p}\left(t\right)\\ C_{p}\left(t\right) \end{array}\right).$$ (8)

Because

$$W_{p}=N_{p}+C_{p},$$ (9)

rewrite Eq (8) in terms of *C*_*p*_ and *W*_*p*_,

$$\left(\begin{array}{c} \frac{dC_{p}\left(t\right)}{dt}\\ \frac{dW_{p}\left(t\right)}{dt} \end{array}\right)=\;\left[\begin{array}{c} -\;(k_{E}+{k^{\prime }}_{f}+k_{D}+k_{f})\\ 0 \end{array}\begin{array}{c} k_{E}+{k^{\prime }}_{f}\\ -k_{T} \end{array}\right]\left(\begin{array}{c} C_{p}\left(t\right)\\ W_{p}\left(t\right) \end{array}\right).$$ (10)

Therefore,

$$\frac{dC_{p}\left(t\right)}{dW_{p}\left(t\right)}=\frac{\;(k_{E}+{k^{\prime }}_{f}+k_{D}+k_{f})C_{p}\left(t\right)}{k_{T}W_{p}\left(t\right)}-\frac{k_{E}+\;{k^{\prime }}_{f}}{k_{T}},$$ (11)

from which we can get the analytical solution of *C*_*p*_ in terms of *W*_*p*_

$$C_{p}\left(W_{p}\left(t\right)\right)=C_{0}{\left(\frac{W_{p}\left(t\right)}{W_{0}}\right)}^{\frac{\;k_{E}+{k^{\prime }}_{f}+k_{D}+k_{f}}{k_{T}}}+W_{p}\left(t\right)\frac{\;k_{E}+{k^{\prime }}_{f}}{\;k_{E}+{k^{\prime }}_{f}+k_{D}+k_{f}-k_{T}}\left(1-{\left(\frac{W_{p}\left(t\right)}{W_{0}}\right)}^{\frac{\;k_{E}+{k^{\prime }}_{f}+k_{D}+k_{f}-k_{T}}{k_{T}}}\right),$$ (12)

Because of Eq (5),

$$C_{p}\left(t\right)=C_{0}e^{-\left(\;k_{E}+{k^{\prime }}_{f}+k_{D}+k_{f}\right)t}+W_{0}\frac{\;k_{E}+{k^{\prime }}_{f}}{k_{E}+{k^{\prime }}_{f}+k_{D}+k_{f}-k_{T}}\left(e^{-k_{T}t}-e^{-(k_{E}+{k^{\prime }}_{f}+k_{D}+k_{f})t}\right).$$ (13)

Similarly,

$$N_{p}\left(t\right)=N_{0}e^{-\left(\;k_{E}+{k^{\prime }}_{f}+k_{f}\right)t}+W_{0}\frac{k_{f}}{k_{E}+{k^{\prime }}_{f}+k_{f}-k_{T}}\left(e^{-k_{T}t}-e^{-(k_{E}+{k^{\prime }}_{f}+k_{f})t}\right).$$ (14)

To determine the transfer rates of ${k^{\prime }}_{f}$, *k*_*f*_ in mitosis, we considered the process of cell cycle. Because of the nature of the quasi-steady state in which stable proportionality of each phase of the cell cycle exists, we can determine during the doubling time of D = 24 hours, the duration of G1/S (*F*_G1/S_) takes about 20% of the time and G2/M (*F*_G2/M_), in which cells have roughly two fold of cellular content compared to G1/S, takes about 80% of the time, based on published the FACS profile[78] [79] [80]. The duration of mitosis (*F*_M_) of drosophila cells takes around 1 hour. Kc167 cells have relatively large nuclei with the ratio of the diameters between the nucleus and the cell equals to *r*_*nc*_ = 4:5. Therefore, for every hour the cytoplasmic RNA that diffuses into the nucleus at the end of telophase is

$$\frac{\left(r_{nc}{}^{3}\times 2\right)}{D\times \left(F_{\text{G}1/\text{S }}\times 1+F_{\text{G}2/\text{M }}\times 2\right)}\left(N+C\right)=\frac{2r_{nc}{}^{3}\left(N+C\right)}{D(F_{\text{G}1/\text{S }}+2F_{\text{G}2/\text{M }})}.$$ (15)

And for every hour the nuclear RNA that diffuses into the cytoplasm at the beginning of M phase is

$$\frac{2N}{D\times \left(F_{\text{G}1/\text{S }}\times 1+\left(F_{\text{G}2/\text{M }}-F_{\text{M }}\right)\times 2\right)}=\frac{2N}{D(F_{\text{G}1/\text{S }}+2F_{\text{G}2/\text{M }}-2F_{\text{M })}}.$$ (16)

To obtain the kinetic rates of nucleocytoplasmic dynamics, we considered four attributes that ought to be satisfied,

1. mRNA dynamics of the nuclear compartment.
  Eq (14).
2. mRNA dynamics of the cytoplasmic compartment.
  Eq (13).
3. quasi-steady-state of the cytoplasmic compartment,
  Eq (7).
4. The relationship between cytoplasmic decay rate and overall turnover rate,
  $$k_{D}C_{0}=k_{T}W_{0}.$$ (17)

RNA-seq experiments render an over-dispersed non-Gaussian distribution for technical noise [81]. To adjust for this effect, we performed local polynomial regression fitting with the coefficient of variation (CV) with the mean value (m) for all the data points from the two biological replicates using the function loess in the package of stats in R, by which we generated function L that represents the numerical correspondence of loess.

CV=L(m). (18)

Thus, taking differential dispersions at individual time points into account we compute the difference on logarithmic scale, and minimized the corresponding four-component fitting gradient by least square.

$$\Theta =[\frac{1}{\text{n}}\overset{n}{\underset{i=1}{\Sigma }}\left(L(N_{m}\left(t_{i}\right)\right)log\frac{N_{m}\left(t_{i}\right)}{N_{p}\left(t_{i}\right)}{)}^{2},\frac{1}{\text{n}}\overset{n}{\underset{i=1}{\Sigma }}\left(L(C_{m}\left(t_{i}\right)\right)log\frac{C_{m}\left(t_{i}\right)}{C_{p}\left(t_{i}\right)}{)}^{2},L(N_{0})L(C_{0}){\left(log\frac{(k_{E}+{k^{\prime }}_{f})N_{0}}{\left(k_{D}+k_{f}+g\right)C_{0}}\right)}^{2},L(W_{0})L(C_{0}){\left(log\frac{k_{T}W_{0}}{(k_{D}C_{0}}\right)}^{2}]$$ (19)

We used the Levenberg—Marquardt algorithm to fit experimental data using the ModFit function in the package of FME in R.

### Robustness of the modeling

To investigate the robustness of modeling in relation to the depth of sequencing, we randomly down-sampled the sequencing reads to only 25 percent of the original number. In this case, the number of genes that were detected in all samples after length correction was reduced to 3519, of which 3403 pass the threshold of *r*^2^ > 0.8. The consistency of the modeled rates was very high between the original and the down-sampled data, indicating that the performance of modeling is quite resilient to the reduction of sequencing depth (S4A–S4C Fig).

### Analysis of kinetic rates in relation with transcript length, RBP binding and chromatin states

Annotations from BioMart (http://www.biomart.org/) for *Drosophila* melanogaster genome BDGP5 were used for these analyses.

For every gene, the length of transcript, intron, 3’UTR and 5’UTR and the number of exons were defined by the maximal values in each category from BioMart annotations. Spearman’s and Pearson’s correlations were calculated to associate length with kinetic rates.

RBP binding data were retrieved from Stoiber et al [31], in both binary form (bound and unbound) and quantitative form (binding scores of the bound genes). To compare the kinetic difference between transcripts bound and unbound by a specific RBP, binary information was used to stratify the genes into two groups and two-sided Wilcoxon rank sum test were performed to calculate the statistical significance that was adjusted with the Holm—Bonferroni correction for multiple comparisons. Because RBP binding may have been underestimated for the 30% lowest expressed transcripts (Figure S2 of [31]), we restricted this analysis to the 70% highest expressed genes (4457 genes total). Overlapping binding of RBP X and RBP Y were calculated as

$$Overlap\left(X,Y\right)=\frac{No.\;of\;genes\;bound\;by\;X\;and\;Y}{No.\;of\;genes\;bound\;by\;X}$$

$$Overlap\left(Y,X\right)=\frac{No.\;of\;genes\;bound\;by\;X\;and\;Y}{No.\;of\;genes\;bound\;by\;Y}$$

To compute the pausing index of Pol II, we used Pol II Chip-seq data of *Drosophila* Kc167 cells from the modENCODE project (DCCid: modENCODE_5569), and calculated the ratio of Pol II signal within 200 bp around TSS and Pol II signal from 201bp to the end of the gene, similar to previous studies [33]. To investigate the relationship between binding strength of a specific RBP and kinetic rates, Spearman’s correlations were calculated.

Chromatin states data were from Filion et al [47] and Kharchenko et al [51]. For every gene, the coordinates of the most 5’ TSS and the most 3’ TTS from BioMart annotation were defined as the TSS and TTS of the gene, for which corresponding chromatin states were assigned. kinetic rates associated with each chromatin state were compared by ANOVA and the statistical significance was calculated with Tukey’s range test.

### Gene ontology analysis

GO analysis was performed using the single ranked list method on the Gorilla server ([82], http://cbl-gorilla.cs.technion.ac.il/). Corresponding p values were retrieved and gene ontology processes with p < 10^−11^ for at least one kinetic processes were displayed in a heatmap generated by the ‘pheatmap’ R package.

## Supporting information

S1 Fig Experimental scheme of metabolic labelling and nucleocytoplasmic fractionation. Blue lines represent pre-existing cytoplasmic RNA; green lines represent pre-existing nuclear RNA; purple lines represent newly-synthesized RNA; orange lines represent yeast RNA spike-in. For a detailed description of the experimental procedure see Methods.
(EPS)

S2 Fig Effects of 4sU labelling and fractionation. (**A**) Scatter plot showing the log_10_ transformed FPKM value of gene expression for *Drosophila* Kc167 cells unlabeled *versus* labelled for 480 minutes. (**B**) Scatter plot showing the log_10_ transformed FPKM value of gene expression for unfractionated *Drosophila* Kc167 cells *versus* the sum of the nuclear and cytoplasmic fractions. Red dots in (A) and (B) mark genes that were excluded from computation of kinetic rate constants; red lines indicate the cutoff functions (see also Methods).
(EPS)

S3 Fig Quality controls of experimental data. **(A-D)** Consistency between two independent biological replicates (rep1 and rep2) of the relative abundance of pre-existing RNA for both nuclear **(A, B)** and cytoplasmic **(C, D)** fractions at 0 min **(A, C)** and 450 min **(B, D)** of labeling. **(E)** Time series of the relative abundance of cytoplasmic and nuclear pre-existing RNA relative to the yeast RNA spike-in. Error bars depict standard deviation between two biological replicates. **(F)** Total mRNA abundance (nuclear and cytoplasmic combined) normalized to yeast spike-in as a function of time. Model fitting was used to determine the plateau level (dotted red line) that is eventually reached. **(G, H)** log_2_ of exon over intron ratio of two genes, *Lam* and *Fmo-2*, in both nuclear and cytoplasmic fractions of two biological replicates. **(I)** Time series of the remaining ribosomal RNA after polyA selection on linear scale. Error bars show standard deviation between two biological replicates.
(EPS)

S4 Fig Testing robustness of the model by down-sampling. Sequence reads were randomly down-sampled to 25 percent and modeling was repeated. **(A-C)** show the consistency between rate constants determined from the down-sampled and full datasets.
(EPS)

S5 Fig Features of genes that are not modeled well. Comparison of transcription rates **(A)** and intron lengths **(B)** of genes that are modeled well and not modeled well, using r^2^ = 0.8 as cutoff.
(EPS)

S6 Fig Additional analyses of links between RBP binding and kinetic rates. **(A-F)** Same analysis as in Fig 3A, 3C and 3D, but for the 50% shortest **(A-C)** and 50% longest **(D-F)** genes. **(G)** Boxplot showing log_2_ transformed export rates of intron-less and intron-containing genes. Central horizontal lines represent median values; box margins represent 25th and 75th percentiles, and whiskers represent the 5th percentile and the 95th percentile. **(H)** Boxplot showing 3’UTR length of intron-less and intron-containing genes. **(I)** Log2 transformed ratio between the number intron-less genes and the number of intron-containing genes, for mRNAs bound by the indicated RBPs according to Stoiber et al [31]. **(J)** Heatmap showing pairwise overlap of mRNA binding by RBPs, sorted by hierarchical clustering. Overlap was calculated as the ratio between the number of the overlapping mRNAs and the number of all mRNAs bound by a particular RBP.
(EPS)

S7 Fig Associations of chromatin states and kinetic rates of mRNA using an alternative, 9-state chromatin model [51]. **(A)** transcription rates of genes divided by their chromatin states at their transcription start sites (TSS). Whiskers represent the 5th percentile and the 95th percentile; **(B)** Between each pair of chromatin states at TSS, a line is drawn if there is statistical difference (p <0.01, Tukey’s range test, ANOVA); **(C, D)** same as **(A, B)**, but for chromatin states at transcription termination sites (TTS). Similar analyses are displayed for nuclear export **(E-H)** and cytoplasmic decay **(I-L)**.
(EPS)

S1 Table Kinetic rates and goodness of fit. Excel spreadsheet. As indicated by the header, for two biological replicates, the table contains the rate of transcription (FPKM/min), export (1/min) and cytoplasmic decay (1/min) for each gene, as well as the goodness of fit which is measured by the coefficient of determination (*r*^2^), for both nuclear and cytoplasmic fractions.
(XLSX)

S2 Table Complete table of GO processes in relation with kinetic rates. The layout of the table is identical to that of Fig 5D.
(XLSX)
